# Janus kinase 3 regulates adherens junctions and epithelial mesenchymal transition through β-catenin

**DOI:** 10.1074/jbc.M117.811802

**Published:** 2017-08-17

**Authors:** Jayshree Mishra, Jugal Kishore Das, Narendra Kumar

**Affiliations:** From the Department of Pharmaceutical Sciences, ILR College of Pharmacy, Texas A&M Health Science Center, Kingsville Texas 78363

**Keywords:** adherens junction, β-catenin, epithelial–mesenchymal transition (EMT), JAK, mucosal immunology, barrier functions, protein structure function

## Abstract

Compromise in adherens junctions (AJs) is associated with several chronic inflammatory diseases. We reported previously that Janus kinase 3, a non-receptor tyrosine kinase, plays a crucial role in AJ formation through its interaction with β-catenin. In this report, we characterize the structural determinants responsible for Jak3 interactions with β-catenin and determine the functional implications of previously unknown tyrosine residues on β-catenin phosphorylated by Jak3. We demonstrate that Jak3 autophosphorylation was the rate-limiting step during Jak3 trans-phosphorylation of β-catenin, where Jak3 directly phosphorylated three tyrosine residues, *viz.* Tyr^30^, Tyr^64^, and Tyr^86^ in the N-terminal domain (NTD) of β-catenin. However, prior phosphorylation of β-catenin at Tyr^654^ was essential for further phosphorylation of β-catenin by Jak3. Interaction studies indicated that phosphorylated Jak3 bound to phosphorylated β-catenin with a dissociation constant of 0.28 μm, and although both the kinase and FERM (Band 4.1, ezrin, radixin, and moesin) domains of Jak3 interacted with β-catenin, the NTD domain of β-catenin facilitated its interactions with Jak3. Physiologically, Jak3-mediated phosphorylation of β-catenin suppressed EGF-mediated epithelial–mesenchymal transition and facilitated epithelial barrier functions by AJ localization of phosphorylated β-catenin through its interactions with α-catenin. Moreover, loss of Jak3-mediated phosphorylation sites in β-catenin abrogated its AJ localization and compromised epithelial barrier functions. Thus, we not only characterize Jak3 interaction with β-catenin but also demonstrate the mechanism of molecular interplay between AJ dynamics and EMT by Jak3-mediated NTD phosphorylation of β-catenin.

## Introduction

An adherens junctions (AJ)^3^ is a multimolecular protein complex that connects neighboring cells, thereby maintaining epithelial tissue architecture (1, 2). AJs are created through extracellular homotypic protein–protein interactions between single-pass trans-membrane cadherin molecules. Intracellularly, these cadherins bind to the catenin family of proteins that link to dense actin filament–based cortical structures (3). The plasticity of AJ remodeling is essential for several physiological processes, including epithelial–mesenchymal transition (EMT), morphogenesis, proliferation, migration, differentiation, and mucosal restitution (4–6). Compromised AJ plasticity is associated with several chronic inflammatory diseases, such as inflammatory bowel disease, colonic dysbiosis, obesity, and metabolic syndrome (4, 7, 8).

β-Catenin, originally identified as AJ-associated protein, is a member of the catenin family of proteins that links the cytoplasmic domain of cadherins to actin cytoskeleton–bound α-catenin (9). Structurally, β-catenin has three main regions: the N-terminal domain (NTD); the central core, which consists of 12 armadillo (ARM) repeat subdomains; and the C-terminal domain (CTD). Each ARM repeat consists of three α helices arranged in a compact superhelix (10). A positively charged groove spans the entire ARM region that creates the binding surface for the majority of β-catenin partners, many of them critical for AJ formation and Wnt signaling (9). NTD and CTD sequences of β-catenin are less conserved than ARM and do not form stable structures by themselves (10). The NTD of β-catenin connects E-cadherin to α-catenin, a key regulator of the actin cytoskeleton (11). Studies indicate that the unstructured NTD and CTD tails interact with the ordered armadillo repeat domain in a dynamic and variable manner, thereby stabilizing the molecule. Traditionally, serine/threonine phosphorylation of β-catenin has been implicated in AJ remodeling (12); however, the structure–function implications of tyrosine phosphorylation of β-catenin in general and the NTD of β-catenin in particular in AJ remodeling are not known.

Jak-3, a member of the Janus kinase family of non-receptor tyrosine kinases, mediates signals initiated by cytokines through interactions with the common γ chain of several cytokine receptors (13). Although Jak3 plays an essential role in preventing intestinal inflammation and associated obesity, metabolic syndrome, (8), and colitis (7), abnormal activation of Jak3 is associated with human malignancies (15, 16), indicating that a tight balance of its activity is essential for normal physiology (17). Like other Jak proteins, Jak3 contains seven Jak homology (JH) domains; JH3–JH4 regions have homology with SH2 domains, and JH6–JH7 domains have homologies with the FERM (Band 4.1, ezrin, radixin, and moesin) domain, found in molecules such as Band 4.1, ezrin, radixin, and moesin (15). The FERM domain of Jak3 not only mediates intermolecular interactions with the cytokine receptor (18) and adapter protein Shc (16, 19) but also facilitates intramolecular binding to the SH2 domain of Jak3, thereby maintaining a close molecular conformation (20). Jak3–FERM domain binding to Shc mediates recruitment of the phosphatases SHP2 and PTP1B to the Jak3–Shc complex, which leads to dephosphorylation of Jak3 (19). We reported previously that Jak3 regulates intestinal inflammation and predisposition to colitis through interaction with and AJ localization of β-catenin (21). However, the mechanism of β-catenin interaction with Jak3 was unknown. In this report, we delineate the structural determinants responsible for β-catenin interactions with Jak3 and demonstrate the molecular mechanism of Jak3-mediated β-catenin phosphorylation–dependent regulation of AJ plasticity and EMT.

## Results

### Recombinant Jak3 trans-phosphorylates β-catenin

**Figure 1.**
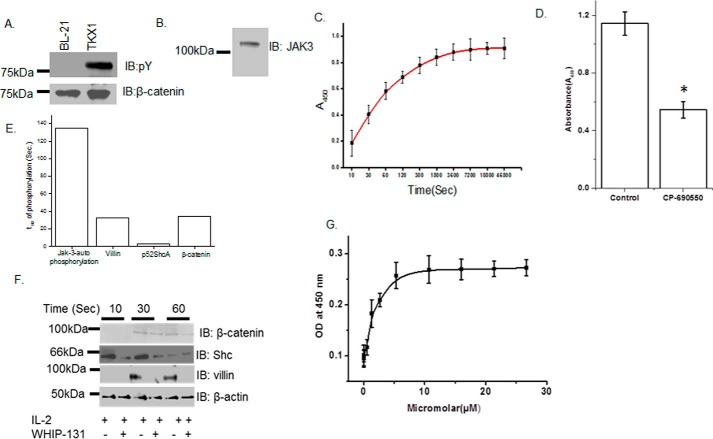
**Recombinant Jak3 trans-phosphorylates the adherent junction protein β-catenin.** *A* and *B*, the non-phosphorylated and phosphorylated forms of the β-catenin protein were produced using BL21 and TKX1 expression system as reported before (20) and were confirmed through IB using phosphotyrosine (*A*, *top panel*), β-catenin (*A*, *bottom panel*) and Jak3 (*B*) antibodies. *C*, changes in tyrosine trans-phosphorylation of β-catenin by Jak3 were detected using a β-catenin–coated 96-well microtiter plate and induction of phosphorylation by addition of activated (P) Jak3 (US Patent 9739779) (14, 20, 49), where P-Jak3-WT alone and β-catenin alone were taken as controls. The phosphorylation was detected as reported before (19, 20). Curve fitting was done as reported before (19) using the Hyperbol-fit program in MicroCal Origin to calculate *t*_½_. *D*, similar experiments were performed as in *C* but in the presence of the Jak3 inhibitor CP-690505 and with a fixed reaction time of 5 min. *E*, comparison of *t*_½_ of Jak3-mediated transphosphorylation of villin (20), p52ShcA (19), and β-catenin. *F*, HT-29 CL-19a cells were treated with IL-2 in the presence or absence of the Jak3 inhibitor CP-690550 for the indicated time, followed by IB using cell lysates from treated cells and the indicated antibodies. *G*, the binding kinetics of Jak3 interactions with β-catenin were determined by a pairwise binding assay, followed by analysis of the data using Hyperbol-fit as reported before (19). *OD*, optical density. *A*, *B*, and *F*, blots are representative of *n* = 3 experiments. *C*, *D*, and *G*, values are mean ± S.E. *, *p* < 0.05, indicating statistically significant differences from the control; *n* = 3 experiments.

Previously we reported that Jak3 facilitated intestinal mucosal barrier functions through its interactions with the adherens junction protein β-catenin (7, 18, 22). To determine the mechanism of Jak3 interaction with β-catenin and the structural determinants that regulate these interactions, we expressed and purified the non-phosphorylated and phosphorylated (P) forms of His-tagged β-catenin–WT using the BL-21 and TKX1 expression systems, respectively (Fig 1*A*). We also expressed and purified the non-phosphorylated form of GST-tagged Jak3-WT using BL-21 expression systems (Fig 1*B*). Because recombinant Jak3-WT autophosphorylates in a time-dependent manner, with a *t*_½_ (the time taken to reach half of the maximum phosphorylation) of autophosphorylation of 135 s (20), we determined whether autophosphorylated Jak3-WT could trans-phosphorylate the non-phosphorylated forms of β-catenin. Fig. 1*C* shows that Jak3 trans-phosphorylated recombinant β-catenin in a time-dependent manner, with a *t*_½_ of trans-phosphorylation of 34 s. To further confirm the trans-phosphorylation of β-catenin by Jak3, the kinase reaction was carried out in the presence of a previously reported (20) Jak3 inhibitor, CP-690550. Fig. 1*D* shows that CP-690550 inhibited phosphorylation of β-catenin by Jak3. Because the slowest step in a reaction is considered rate-limiting, these results showed that autophosphorylation of Jak3 was the rate-limiting step during tyrosine phosphorylation of β-catenin by Jak3, where β-catenin was a direct substrate for Jak3. We reported previously that recombinant Jak3 also phosphorylates the cytoskeletal protein villin and the adapter protein p52ShcA with a *t*_½_ of transphosphorylation of 33 s (20) and 3.2 s (19), respectively, indicating that Jak3-mediated ShcA phosphorylation precedes villin or β-catenin phosphorylation. Confirmation of this through comparison of Jak3-mediated phosphorylation in a human epithelial cell model shows that activation by IL-2 led to time-dependent phosphorylation of p52Shc, villin, and β-catenin, which were specifically inhibited by the Jak3-inhibitor CP-690550, whereas Jak3-mediated phosphorylation of p52ShcA preceded the phosphorylation of villin and β-catenin (Fig. 1, *E* and *F*). Because β-catenin was a direct substrate for Jak3, we determined the kinetics of β-catenin binding to Jak3. Pairwise binding studies showed that Jak3-WT interacted with β-catenin in a dose-dependent manner with a *K_d_* of 0.28 μm (Fig. 1*G*).

### Jak3 phosphorylates Tyr^30^, Ty^r64^, and Tyr^86^ in the NTD of β-catenin

**Figure 2.**
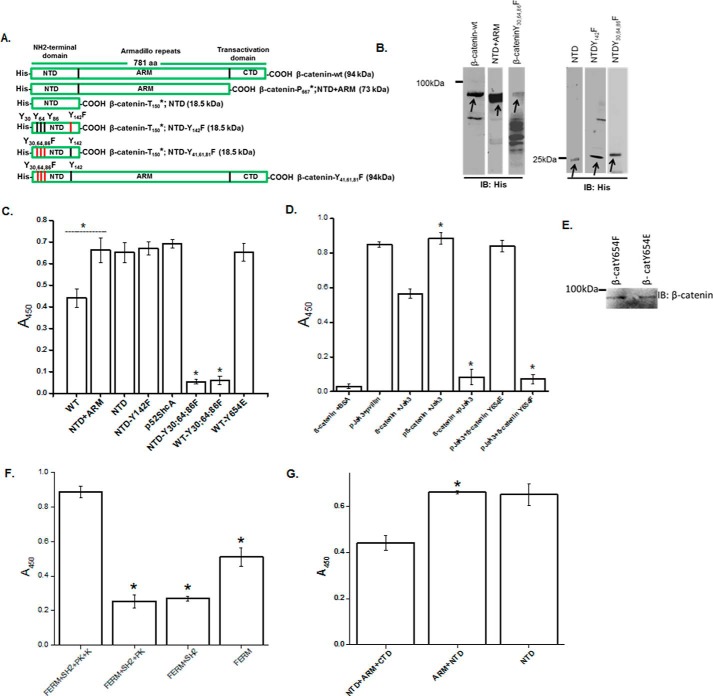
**Characterization of structural determinants responsible for Jak3 interactions with β-catenin.** *A*, schematic of His–β-catenin–WT and mutants. *B*, His-tagged protein β-catenin–WT and these mutants were expressed and purified as in Fig. 1*A* and confirmed through Western blot analysis of the expressed proteins using anti-His antibody. *Arrows* indicate recombinant protein expression. *C*, transphosphorylation experiments were performed using the WT and indicated mutants of β-catenin as substrate. Phosphorylation was induced as in Fig. 1*C*, where P-Jak3-WT alone or β-catenin or its mutants alone were used as controls. *D*, direct interactions between Jak3-WT and β-catenin or its mutants were determined as in *C*, where direct interactions between β-catenin and Jak3 mutants were determined as in Fig. 1*G* using BSA and villin as negative and positive controls, respectively. *E*, Western blot analysis of the indicated purified recombinant proteins of β-catenin were done using β-catenin antibody. *F*, direct interactions between β-catenin–WT and the indicated mutants of Jak3 were determined as in *D. G*, direct interactions between Jak3-WT and the indicated mutants of β-catenin were determined as in *D*. The blots in *B* and *E* are representative of *n* = 3 experiments. *C*, *D*, *F*, and *G*, values are mean ± S.E. *, *p* < 0.05, indicating statistically significant differences from β-catenin–WT (*C*, *D*, and *G*) or Jak3-WT (*F*); *n* = 3 experiments.

Because β-catenin not only interacted with but was also phosphorylated by Jak3, we determined the structural determinants of β-catenin phosphorylated by Jak3. Fig. 2*A* shows a schematic of β-catenin and its mutants. The wild type and these mutant proteins of β-catenin were expressed and purified using the BL21 expression system (Fig. 2*B*) and were used as substrates for an *in vitro* kinase assay using autophosphorylated Jak3 as an enzyme. Fig. 2*C* shows that Jak3 trans-phosphorylated β-catenin; however, deletion of the CTD of β-catenin resulted in a 1.5-fold increase in tyrosine phosphorylation. Moreover, deletion of both the ARM domain and CTD did not change phosphorylation by Jak3, indicating that Jak3-mediated phosphorylation of β-catenin was due to tyrosine residues present in the NTD of β-catenin. Because the NTD of β-catenin has four tyrosine residues, and we reported previously that, in human intestinal epithelial cells, Jak3 does not associate with Tyr^142^-phosphorylated β-catenin (7), using an *in vitro* kinase assay, we determined whether mutation of Y142F of β-catenin has an effect on Jak3-mediated tyrosine phosphorylation of the β-catenin NTD. Fig. 2*C*, *fourth column*, shows that mutation of Y142F indeed did not change the phosphorylation of the NTD of β-catenin, indicating that Jak3 did not phosphorylate Tyr^142^ of β-catenin. To determine the number of tyrosine residues of β-catenin phosphorylated by Jak3, we analyzed the tyrosine residues other than Tyr^142^ present in the NTD domain of β-catenin and the contribution of these toward Jak3-mediated phosphorylation of β-catenin. Comparative analysis of these using methods reported previously (19) and p52ShcA as a positive control showed that Jak3 phosphorylated three tyrosine residues, *viz.* Tyr^30^, Tyr^64^, and Tyr^86^, in the NTD of β-catenin (Fig. 2*C*). To further confirm Tyr^30^, Tyr^64^, and Tyr^86^ as Jak3-mediated tyrosine phosphorylation sites in β-catenin, triple mutation studies using either the NTD alone (Fig. 2*C*, *sixth column*) or full-length β-catenin (Fig. 2*C*, *seventh column*) showed that mutation of all three resulted in complete loss of Jak3-mediated β-catenin phosphorylation (Fig. 2*C*).

### Tyrosine phosphorylation influences β-catenin interactions with Jak3

Because the CTD of β-catenin partially inhibited Jak3-mediated phosphorylation of β-catenin, we determined the effects of tyrosine phosphorylation on the interactions between Jak3 and β-catenin. Fig. 2*D* shows that the non-phosphorylated form of β-catenin interacted with non-phosphorylated Jak3; however, tyrosine phosphorylation of β-catenin (P–β-catenin) increased the interactions between Jak3 and P–β-catenin by 1.6-fold. Surprisingly, non-phosphorylated forms of β-catenin minimally interacted with the phosphorylated forms of Jak3, indicating that prior phosphorylation at the tyrosine residues of β-catenin was necessary for enhanced interaction and further phosphorylation by P-Jak3. Because we reported previously that, in human IECs, Jak3-associated β-catenin had phosphorylation at Tyr^654^ of the CTD (7), and Fig. 1 showed that the non-phosphorylated CTD of β-catenin inhibited NTD phosphorylation by Jak3, using a phosphomimetic (Y654E) approach, we determined whether prior phosphorylation of β-catenin at Tyr^654^ was necessary for the enhanced Jak3 interactions and further phosphorylation of β-catenin. Fig. 2*D* shows that the inhibitory effects of non-phosphorylated β-catenin interactions with P-Jak3 were reversed by using the phosphomimetic (Y654E) form of non-phosphorylated, full-length β-catenin. Moreover, phosphomimetic (Y654E) full-length β-catenin also overcame the inhibitory effects of CTD toward Jak3-mediated phosphorylation, which was equivalent to the maximum phosphorylation level of NTD by P-Jak3 (Fig. 2*C*, *seventh column*). To further confirm the role of Tyr^654^, our data show that tyrosine-phosphorylated forms of the Y654F mutant of β-catenin failed to interact with P-Jak3 (Fig. 2*D*, *seventh column*). Fig. 2*E* shows the Western blot analysis of expression and purification of phosphomimetic (Y654E) and phosphomutant (Y654F) full-length β-catenin.

### The kinase and FERM domains of Jak3 and the NTD domain of β-catenin facilitate Jak3 interactions with β-catenin

Because Jak3 not only interacted with β-catenin but also phosphorylated it, we determined the structural determinants of Jak3 responsible for its interactions with β-catenin. Fig. 2*F* shows that Jak3 interacted with phosphorylated β-catenin; however, deletion of the kinase domain of Jak3 resulted in more than a 4-fold decrease in their interactions. Moreover, further deletion of both the kinase and pseudokinase domains did not affect the level of interaction; however, deletion of the kinase, pseudokinase, and SH2 domains altogether resulted in a 2-fold increase in their interactions. These results indicate that both the kinase and FERM domains of Jak3 were responsible for Jak3 interactions with β-catenin, where the SH2 domain of Jak3 partially inhibited the interactions between the FERM domain of Jak3 and β-catenin. Next we determined whether truncation of β-catenin had an impact on the interactions between β-catenin and P-Jak3. Fig. 2*G* shows that β-catenin interacted with Jak3; however, deletion of the CTD domain increased their interactions, which remained unaffected upon further deletion of ARM domains. This indicated that the NTD domain of β-catenin was necessary and sufficient for its interaction with Jak3.

### Jak3 phosphorylates Tyr^30^, Tyr^64^, and Tyr^86^ of β-catenin in intestinal epithelial cells

**Figure 3.**
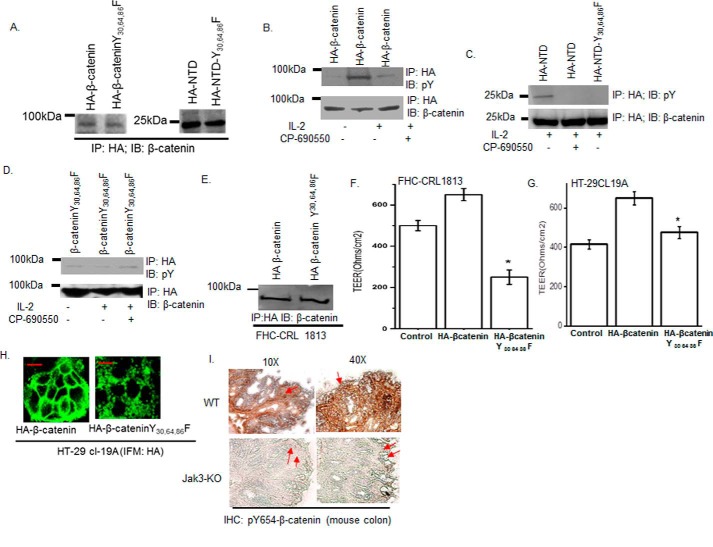
**Jak3 phosphorylation of Tyr^30^, Tyr^64^, and Tyr^86^ in β-catenin facilitates barrier functions.** *A*, Western blot analyses of immunoprecipitates from the cell lysates of stably transfected HT-29 Cl-19A cells were done for the expression of HA-tagged β-catenin or its indicated mutants. *B*, stably transfected cells of HA-tagged β-catenin and its mutant from *A* were treated with IL-2 (50 units/ml) only or with CP-690550 (100 mg/ml). Cell lysates were subjected to IP, followed by IB using the indicated antibodies as reported before (22). *C*, similar experiments were performed as in *B* but using stably transfected cells of HA-NTD or its mutant. *D*, similar experiments were performed as in *B* but using stably transfected cells of HA–β-catenin–Y30F,Y64F,Y86F. *E*, normal epithelial cells, FHC CRL-1813, were transiently transfected with cDNA constructs containing HA-tagged β-catenin–WT or the indicated mutant. IP followed by IB analysis were done using the indicated antibodies and cell lysates from the transfected cells to confirm comparable expression of transfected proteins. *F* and *G*, control (untransfected), HA–β-catenin-, and HA–β-catenin–Y41F,Y61F,Y81F–expressing FHC CRL-1813 (*F*) and HT-29 Cl-19a (*G*) cells were cultured in a 6-transwell plate to confluence, and TEER was measured. Mean TEER values (*n* = 6) are shown. *, *p* < 0.05, indicating statistically significant differences from β-catenin–WT–transfected cells. *H*, AJ localization of β-catenin was determined using IFM in confluent HT-29 Cl-19a cells stably transfected with either HA-β-catenin or HA–β-catenin–Y30F,Y64F,Y86F using anti-HA antibody. *Green* indicates localization of HA-tagged proteins. Note that mutation in β-catenin at Y30F,Y64F,Y86F disrupts its AJ localization, as denoted by punctate *green* staining. *Scale bar* = 14 μm. *I*, IHC staining for the localization of Tyr^654^-phosphorylated β-catenin in colonic mucosa of WT and Jak3 KO mice was performed using the protocol described under “Experimental procedures.” *Red arrows* indicate the differences in localization of Tyr^654^-phosphorylated β-catenin in the absence of Jak3. Note that lack of Jak3 results in loss of localization of Tyr^654^-phosphorylated β-catenin at cellular junctions. *A–E*, the blots shown are representative (*n* = 3). *H* and *I*, the data shown are representative (*n* = 6/group).

Because P–β-catenin not only interacted with P-Jak3, but Jak3 also phosphorylated three tyrosine residues in the NTD of β-catenin, we determined the physiological significance of these in a human intestinal epithelial cell (IEC) model. Human IECs, HT-29 Cl-19A, were stably transfected either with pCDNA–HA–β-catenin–WT, pCDNA–HA–β-catenin–Y30F,Y64F,Y86F, pCDNA-HA-NTD, or pCDNA-HA-NTD-Y30F,Y64F,Y86F, and clones expressing comparable amounts of recombinant proteins were selected (Fig. 3*A*). To determine whether Jak3 had an effect on the phosphorylation of specific tyrosine residues on β-catenin, these stably transfected cells were treated with IL-2, and cell lysates were immunoprecipitated with HA and immunoblotted either with phosphotyrosine or β-catenin antibody. We reported previously that treatment with IL-2 led to activation (7, 8, 18–20) of Jak3 in IECs, which facilitated Jak3 interactions with its substrate (22), including β-catenin (7). We showed that IL-2 activation led to tyrosine phosphorylation of both β-catenin (Fig. 3*B*) and the NTD of β-catenin (Fig. 3*C*) but not of NTD with a triple tyrosine mutation (NTD-Y30F,Y64F,Y86F) (Fig. 3*C*). Moreover, treatment of IL-2 stimulated IECs with a Jak3 inhibitor led to decrease in β-catenin–WT phosphorylation (Fig. 3*B*) and complete loss of β-catenin–NTD phosphorylation, confirming (Fig. 3*C*) that Jak3 phosphorylates the NTD domain of β-catenin, where Tyr^30^, Tyr^64^, and Tyr^86^ are the tyrosine residues phosphorylated by Jak3 in a human IEC. Moreover, cells with mutation of Tyr^30^, Tyr^64^, and Tyr^86^ to Phe in full-length β-catenin did not show a change in phosphorylation either by IL-2 stimulation or by Jak3 inhibition in IL-2–stimulated cells (Fig. 3*D*), further confirming Tyr^30^, Tyr^64^, and Tyr^86^ as the Jak3-mediated phosphorylation sites in full-length β-catenin.

### Jak3-mediated tyrosine phosphorylation of Tyr^30^, Tyr^64^, and Tyr^86^ of β-catenin facilitates epithelial barrier functions

Because β-catenin facilitates AJ formation, which in turn regulates the barrier functions, we determined the physiological consequence of Jak3-mediated phosphorylation of β-catenin on AJ formation and associated barrier functions in both cancerous (HT-29 cl-19a) and normal (FHC-CRL-1813) human IEC models transfected with either HA–β-catenin or HA–β-catenin–Y30F,Y64F,Y86F (Fig. 3, *A* and *E*, respectively). Fig. 3, *F* and *G*, shows that expression of HA–β-catenin increased the trans-epithelial electrical resistance (TEER) in both IEC models, whereas expression of the Jak3-mediated phosphorylation (Y30F,Y64F,Y86F) mutant of HA–β-catenin led to a reversal of these effects, indicating that the use of transformed IEC did not influence the physiological significance of Jak3-mediated phosphorylation of β-catenin. To further confirm that these physiological effects were due to loss of Jak3-mediated tyrosine phosphorylation sites in β-catenin and associated impairment of AJ formation, using confocal microscopy, we determined the AJ localization HA–β-catenin and its triple mutation counterparts. Fig. 3*H* shows that HA–β-catenin mostly localized to the AJ in IECs, as indicated by strong β-catenin staining at the cellular junctions. However, mutation of Jak3-mediated tyrosine phosphorylation sites (Tyr^30^, Tyr^64^, and Tyr^86^) in β-catenin led to a substantial decrease in β-catenin localization to the AJ, as indicated by punctate β-catenin staining at the cellular junctions. Because prior phosphorylation at Tyr^654^ of β-catenin was essential for its interactions and subsequent phosphorylation by Jak3 (Fig. 2*C*) that led to AJ localization of phosphorylated-β-catenin, to demonstrate the *in vivo* significance of these findings, we determined whether absence of Jak3 affected the AJ localization of Tyr^654^-phosphorylated β-catenin in the mouse colon. Fig. 3*I*, *top panels*, shows that Jak3-expressing WT mice had Tyr^654^-phosphorylated-β-catenin mostly localized to the cellular junctions; however, lack of Jak3 (Fig. 3*I*, *bottom panels*) resulted in loss of junctional localization of Tyr^654^-phosphorylated β-catenin. Moreover, Jak3 KO mice showed diffused and substantially reduced pTyr^654^–β-catenin expression in the colonic epithelium. This further corroborates our earlier reports that lack of Jak3 leads to compromised colonic barrier functions in mice (7, 8).

### Jak3-mediated phosphorylation of β-catenin prevents EMT

**Figure 4.**
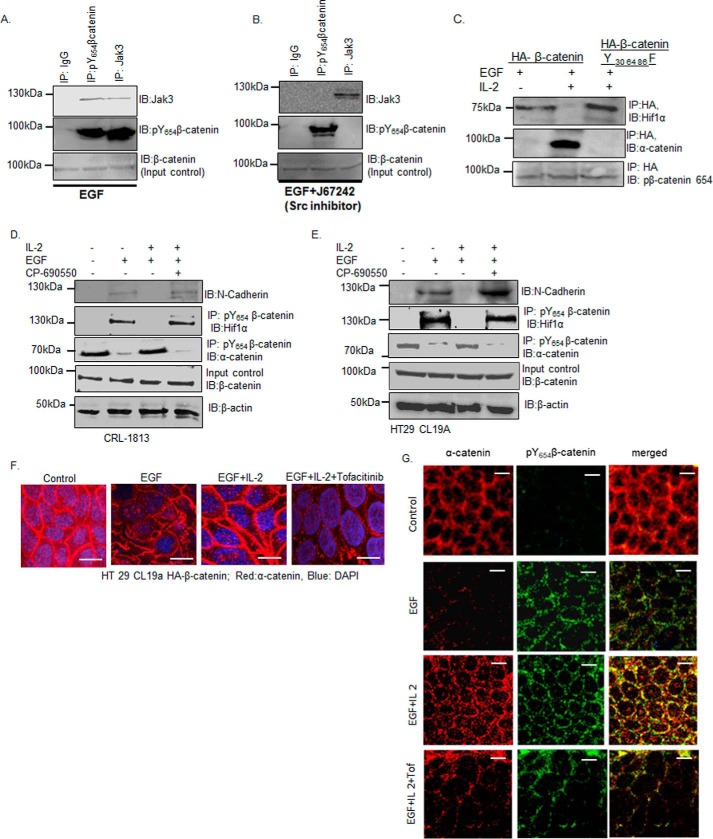
**Phosphorylation of Tyr^30^, Tyr^64^, and Tyr^86^ in β-catenin by Jak3 suppresses EMT.** *A* and *B*, HT-29 CL19a cells were stimulated with either EGF (100 μg/ml) alone (*A*) to facilitate Tyr^654^ phosphorylation of β-catenin or in the presence of the Src inhibitor J67242 (50 mm) (*B*) to prevent Tyr^654^ phosphorylation of β-catenin. Association of Jak3 with Tyr^654^-phosphorylated β-catenin was determined by co-IP using the indicated antibodies. IB using β-catenin antibody was done for input control. *C*, HT-29 CL19a cells expressing either HA–β-catenin or HA–β-catenin–Y30F,Y64F,Y86F were stimulated either with EGF (100 μg/ml) alone or in the presence of IL-2 (50 units/ml). β-Catenin association either with α-catenin as an indicator of AJ integrity or with Hif1α as an indicator of mesenchymal transition was determined using co-IP, followed by IB with indicated antibodies. *D* and *E*, EMT was induced in normal epithelial cells, CRL-1813 (*D*), and in transformed epithelial cells, HT-29 Cl-19a (*E*), by stimulation with EGF-mediated activation of Src kinase as reported before (23), and Jak3-mediated suppression of EMT was determined through simultaneous activation of the cells by EGF and IL-2. Induction of EMT was confirmed by IB for the EMT marker N-cadherin and co-IP for β-catenin association with Hifα. AJ stabilization was confirmed through co-IP for β-catenin association with α-catenin. IB using β-catenin indicates input control, and using β-actin indicates loading control. *A–E*, blots are representative (*n* = 3 experiments). *F* and *G*, HT-29 CL19a cells were stimulated either with EGF alone in the presence of IL-2, or in the presence of IL-2 + the Jak3 inhibitor tofacitinib, and AJ integrity was determined through confocal microscopy analysis of either α-catenin localization alone (*F*) or through its co-localization with Tyr^654^-phosphorylated β-catenin (*G*). Representative images are shown (*n* = 6 experiments). *Green* indicates Tyr^654^-phosphorylated β-catenin, and *red* indicates α-catenin. *Scale bars* = 14 μm.

Src kinase phosphorylates β-catenin at Tyr^654^, which promotes EMT through β-catenin association with Hif1α (23). Because our *in vitro* data showed that prior phosphorylation of β-catenin at Tyr^654^ was necessary for its interaction with and further phosphorylation by Jak3, we first confirmed whether this was the case in human IECs through activation of Src alone, and then we determined whether Jak3-mediated phosphorylation of β-catenin prevented EMT through AJ localization of β-catenin during simultaneous activation of Src and Jak3 in human IECs. Fig. 4, *A* and *B*, shows that co-immunoprecipitation (co-IP) of Tyr^654^-phosphorylated β-catenin with Jak3 was dependent on EGF-induced activation of Src, followed by Src-mediated phosphorylation of Tyr^654^ of β-catenin. For these experiments, IP with IgG represented as negative control, IP followed by IB with pTyr^654^–β-catenin antibody represented a positive control, and IB for total β-catenin was used as an input control. Fig. 4*C*, *bottom panel*, shows that, EGF stimulation of HA-tagged β-catenin led to a similar level of Tyr^654^ phosphorylation of β-catenin; however, co-stimulation with IL-2 led to disruption of interactions between HA-tagged β-catenin and Hif1α, which were reversed by mutation of Jak3-mediated phosphorylation sites (Y30F,Y64F,Y86F) in β-catenin (Fig. 4*C*, *top panel*). Furthermore, EGF also promoted disruption of HA-tagged β-catenin with the AJ protein α-catenin, which was again reversed by mutation of Jak3-mediated phosphorylation sites (Y30F,Y64F,Y86F) in β-catenin (Fig. 4C, *center panel*). To determine that this was not due to transformation-related factors of epithelial cells, Fig. 4*D* shows that activation of CRL-1813cells, a normal epithelial cell line, or HT-29cl-19a cells (Fig. 4*E*), with EGF led to expression of the EMT marker N-cadherin, which was associated not only with co-immunoprecipitation of Tyr^654^-phosphorylated β-catenin with Hif1α but also dissociation of Tyr^654^-phosphorylated β-catenin from the AJ protein α-catenin, indicating a disruption of the AJ. However, simultaneous activation by the Src activator EGF and the Jak3 activator IL-2 led to a reversal of these effects, as indicated by increased association of Tyr^654^-phosphorylated β-catenin with α-catenin, reflecting AJ localization of β-catenin, which was associated with decreased expression of the EMT marker N-cadherin. To further confirm that these effects were due to Jak3 activation, the experiments were performed in the presence of a Jak3 inhibitor (CP690550). Fig. 4, *D* and *E*, shows that inhibition of Jak3 in EGF and IL-2–stimulated cells not only promoted EMT, as reflected by the expression of N-cadherin, but also prevented association of Tyr^654^-phosphorylated β-catenin with the AJ protein α-catenin, indicating AJ destabilization. This indicated that, irrespective of IEC types, Jak3 activation was required for the prevention of Src-mediated EMT in these epithelial cells. Because α-catenin interaction with β-catenin was necessary for AJ localization of α-catenin, and EGF-mediated tyrosine phosphorylation of β-catenin at Tyr^654^ disrupts these interactions, using confocal microscopy, we determined the effects Jak3 activation on α-catenin localization to the AJ. Fig. 4*F* shows that, in control unstimulated cells, α-catenin was mostly localized to cellular junctions. Moreover, stimulation by EGF led to loss of AJ localization of α-catenin, indicating Tyr^654^ phosphorylation of β-catenin. However, these effects were reversed by co-stimulation with EGF and IL-2, as indicated by increased junctional localization of α-catenin. To confirm that these reversals are due to Jak3-mediated β-catenin phosphorylation, the experiments were carried out in the presence of the Jak3 inhibitor tofacitinib. As shown in Fig. 4*F*, *fourth panel*, inhibition of Jak3 led to significant loss of AJ localization of α-catenin, as indicated by punctate α-catenin staining at the cellular junctions. To further confirm that Jak3-mediated changes in α-catenin localization were due to its interactions with β-catenin, using confocal microscopy, we showed that EGF activation of IECs led to loss in junctional localization of α-catenin (Fig. 4*G*, *second row*), which was significantly reversed by co-stimulation of these cells with IL-2 (Fig. 4*G*, *third row*), as indicated by strong yellow staining in the *merged panel*. To confirm that these effects were due to Jak3 activation, these experiments were performed in the presence of Jak3 inhibitor. Fig. 4*G*, *fourth row*, shows that inhibition of Jak3 significantly decreased α-catenin co-localization with Tyr^654^-phosphorylated β-catenin.

**Figure 5.**
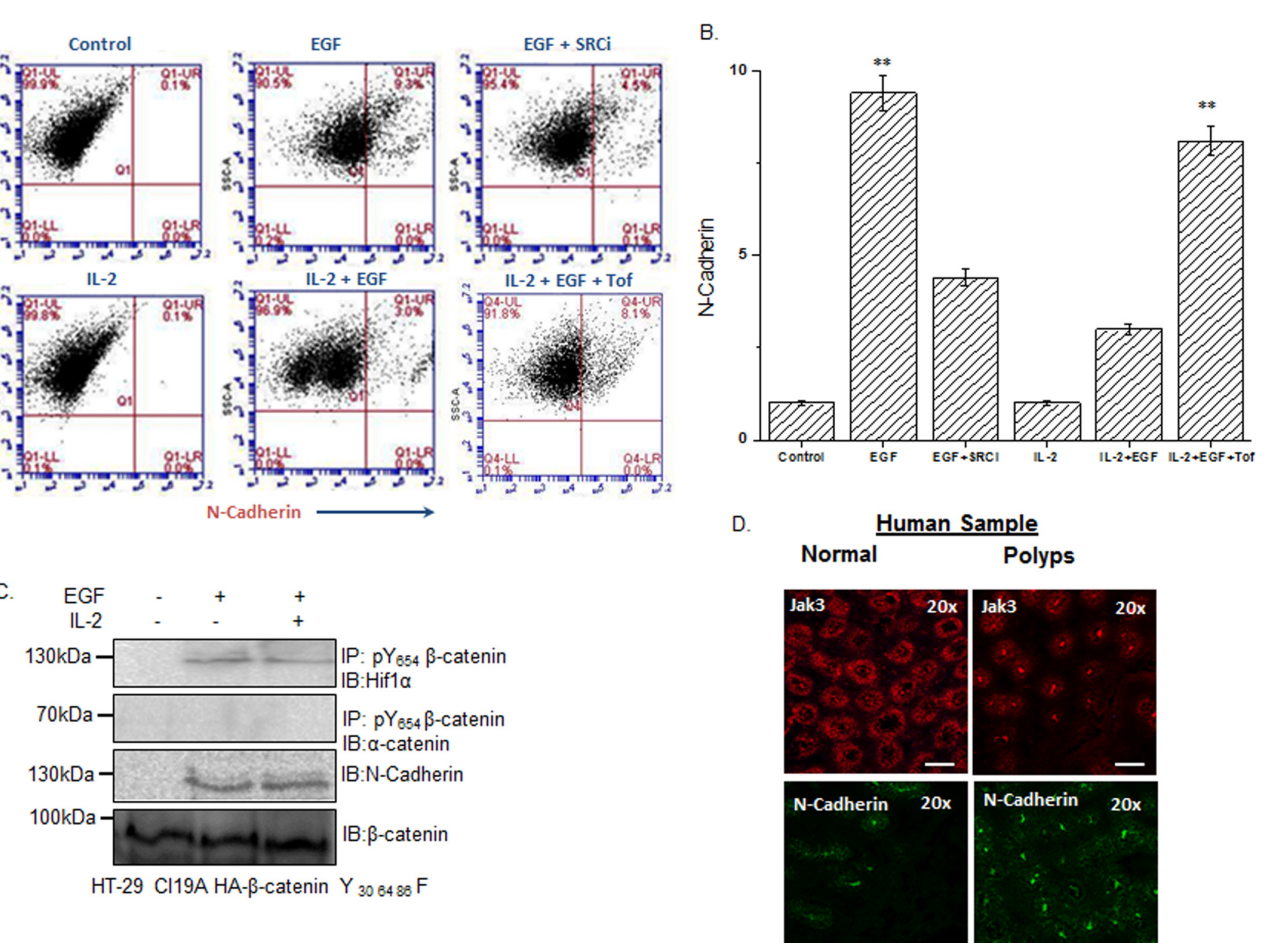
**Loss of Tyr^30^, Tyr^64^, and Tyr^86^ phosphorylation in the NTD domain of β-catenin facilitates EMT.** *A* and *B*, HT-29 Cl-19a cells were either left unstimulated (control) or stimulated with EGF and/or IL-2 alone or in the presence of Src-kinase inhibitor (*SRCi*) or Jak3 kinase inhibitor (*Tof*). Flow cytometry estimation of the cells was performed as detailed under “Experimental procedures” to determine expression of the EMT marker N-cadherin. Note that the *top panels* in *A* confirm EGF stimulation of the EMT marker N-cadherin, whereas the *bottom panels* show reversal of these effects by IL-2–mediated activation of Jak3. *B*, there was a 10-fold increase in expression of the EMT marker by EGF that was suppressed almost 3-fold by IL-2 activation of Jak3. *A*, scatterplots are representative (*n* = 3 experiments). *B*, plots are the average of three experiments. **, indicating a statistically significant difference with control (for EGF) and with IL-2+EGF (for IL-2+EGF+Tof). *C*, stably transfected cells of Jak3-mediated phospho-mutants (Y30F,Y64F,Y86F) of β-catenin in HT-29 Cl-19a cells were either taken unstimulated (control) or stimulated with EGF alone or in the presence of IL-2. Cell lysates were prepared and subjected to either IB alone or IP followed by IB using the indicated antibodies. Although association of β-catenin with α-catenin determined AJ stabilization, expression of the EMT marker N-cadherin and β-catenin association with Hifα determined the induction of EMT. IB using β-catenin antibody demonstrated input controls. Blots are representative (*n* = 3 experiments). *D*, deidentified and discarded colonic tissue samples of human subjects either healthy or having colonic polyps were immunostained either with the EMT marker N-cadherin or our newly identified EMT suppressor Jak3 using procedures detailed under “Experimental procedures.” Images were acquired using a Nikon C1-plus laser confocal microscope, and representative images are shown from each group (*n* = 5). Note the significantly decreased expression and altered localization of the EMT suppressor Jak3 in colonic polyps (*right panels*) compared with healthy tissues (*left panels*). Also note the increased expression of the EMT marker N-cadherin exclusively in colonic polyps, where Jak3 expression was markedly decreased. *Scale bars* = 550 μm.

To quantitate the extent of the effects of Jak3 on Tyr^654^-phosphorylated β-catenin–mediated reversal of EMT, using flow cytometry, we determined the effects of EGF, EGF+IL-2, and EGF+IL-2+tofacitinib on the relative expression of EMT markers. The representative flow data in Fig. 5*A* and the corresponding statistical analysis from three independent experiments in Fig. 5*B* show that there was approximately a 10-fold increase in cells expressing the EMT marker N-cadherin upon EGF-mediated (Fig. 5*B*, *second column*) Src activation (Fig. 5*B*, *third column*). Moreover, IL-2 alone did not influence N-cadherin expression, which was similar to that of the control. However, co-stimulation of IECs by EGF+IL-2 led to a more than 3-fold decrease in cells expressing EMT markers (Fig. 5*B*, *fifth column*), which was correlated with increased AJ localization of β-catenin (Fig. 4). To confirm that these decreases in expression of EMT markers were due to Jak3 activation, the cells were co-stimulated with EGF+IL-2 in the presence of the Jak3 inhibitor tofacitinib. Fig. 5*B*, *sixth column*, shows that the IL-2-mediated decrease in N-cadherin-expressing cells was almost completely restored by inhibition of Jak3. These results were further confirmed by determining the expression of another EMT marker, Hif1-α, using not only the IEC model but also an *in vivo* model of Jak3 KO mouse colon, which showed similar results as N-cadherin (supplemental Figs. S1–S3). To prove the specific role of Jak3-mediated β-catenin-phosphorylation, specific mutations of Jak3-mediated tyrosine phosphorylation residues to phenylalanine (Y30F,Y64F,Y86F) in β-catenin resulted in abrogation of EMT prevention by IL-2 in the presence of EGF, as denoted by loss of responsiveness to IL-2 toward EGF-mediated β-catenin interactions with Hif1-α (Fig. 5*C*, *first panel*), loss of β-catenin interactions with α-catenin (Fig. 5*C*, *second panel*), and expression of the EMT marker N-cadherin (Fig. 5*C*, *third panel*). Taken together, these results indicate that Jak3 phosphorylates the Tyr^30^, Tyr^64^, and Tyr^86^ residues in Tyr^654^-phosphorylated β-catenin, which not only prevents β-catenin association with Hif1α and expression of the EMT marker N-cadherin but also facilitates Tyr^30^-, Tyr^64^-, and Tyr^86^-phosphorylated β-catenin association with α-catenin, thereby reinforcing AJ formation.

In humans, colonic polyps represent a transition state in the progression of normal epithelial cells to cancerous cells, where EMT plays an essential role in this progression, and sustained expression of the EMT marker N-cadherin is frequently reported (24, 25). Because our data showed that Jak3-activation prevented EMT marker expression both in normal and transformed IECs, we determined whether this was also the case in intestinal resected human colonic epithelial polyps. Fig. 5*D*, *left panels*, shows that, compared with healthy epithelial tissues, there was a significant decrease in expression and localization of Jak3 that was associated with increased expression of the EMT marker N-cadherin in colonic polyps. These results show that a decrease in Jak3 expression and altered localization were associated with increased EMT in human colonic polyps.

### Molecular dynamics of Jak3 phosphorylation of β-catenin

**Figure 6.**
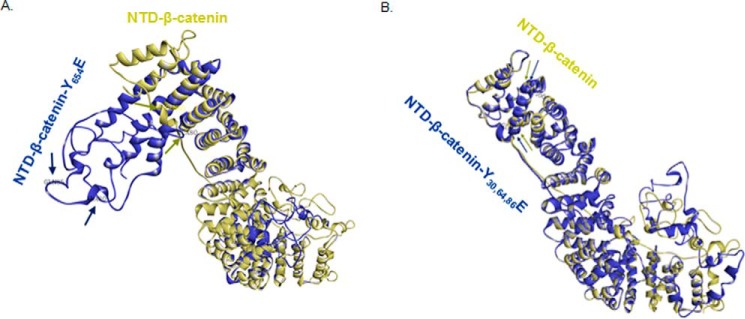
**Molecular dynamics of Jak3-mediated phosphorylation sites in β-catenin.** *A* and *B*, full-length β-catenin was modeled using the Phyre2 modeling server, and structural shifts because of phosphorylation were reconstructed by a phospho-mimetic approach through 3D mutant substitutions of Tyr to Glu either at Y654E (*A*) or at Y30E,Y64E,Y86E (*B*). Structural prediction was done using primary simulations through Phyre2. Energy minimization was done by dynamic simulations using CHARMM Forcefield (Chemistry at Harvard Macromolecular Mechanics) to stabilize the phospho-mutant structures. The conformational shifts in the NTD of β-catenin induced by the Tyr-to-Glu substitutions were evaluated by superimposition of the protein structures and positional determination of residues Ala^80^, Gly^85^, and Val^166^ as markers to evaluate the relative orientation of the NTD of β-catenin. *Arrows* (*gold*, β-catenin–WT; *blue*, β-catenin Y654E) indicate the corresponding positions of Ala^80^, Gly^85^, and Val^166^ in the modeled proteins. Note that superimposed β-catenin–WT (*gold*) on Y30E,Y64E,Y86E (*blue*) in *B* show overlap of the positional markers Gly^85^ and Val^166^ in the structure of the protein, indicating a close resemblance in conformation of the NTD of β-catenin–WT and β-catenin–Y30E,Y64E,Y86E and a reversal of orientation from β-catenin–Y654E in *A*.

Because our results showed that Jak3 phosphorylation of β-catenin not only stabilized AJ but also suppressed EMT, using a phosphomimetic approach for Jak3-mediated β-catenin phosphorylation sites (Y30E,Y64E,Y86E), we performed molecular simulations to compare the structural shifts in wild-type (Tyr) *versus* phosphomimetic mutants (Glu, Y654E and Y30E,Y64E,Y86E) of β-catenin. The simulations on β-catenin–WT and the β-catenin–Y654E mutant show that Y654E induced a significantly changed orientation of the β-catenin NTD (Fig. 6*A*), as indicated by stearic separation of the Gly^85^ and Ala^80^ residues in the NTD of the two superimposed structures of β-catenin–WT (*gold*) and β-catenin–Y654E (*blue*). Because it was reported that native β-catenin mostly localized to AJ through β-catenin–NTD (26, 27) interactions with α-catenin, and phosphorylation of Tyr^654^ facilitates AJ exit of β-catenin (23), we determined whether phosphomimetic mutations of Jak3-mediated tyrosine phosphorylation sites in β-catenin (Y30E,Y64E,Y86E) could preserve the native NTD conformation in β-catenin. The simulations on β-catenin–WT (Fig. 6*B*, *gold*) and β-catenin–Y30E,Y64E,Y86E (Fig. 6*B*, *blue*), followed by stearic superimposition in the structures, show a close resemblance in the NTD, as indicated by the relative positions of Gly^85^ and Val^166^ in the superimposed proteins (Fig. 6*B*). This indicates that Y654E-mediated large shifts in NTD orientations were reversed by simultaneous mutations in Y30E,Y64E,Y86E, which was close to that of native β-catenin. Thus, the Y30E,Y64E,Y86E mutation that mimicked the phosphorylation state of these residues preserved the geometric flexibility of the NTD required for AJ localization of β-catenin.

## Discussion

AJs play a major role in maintaining the integrity of epithelial tissue architecture (1, 2). The plasticity of AJs has several physiological and pathophysiological implications, including morphogenesis, proliferation, migration, and differentiation during mucosal restitution (4, 5), EMT (6), and neoplastic transformation (28). Although compromised AJ plasticity has been reported in several chronic inflammatory diseases (4, 7, 8), the underlying mechanisms are poorly understood. In an effort to understand the mechanism of AJ plasticity, we sought to investigate the tyrosine phosphorylation–mediated functional regulation of an AJ.

Jak3, a non-receptor tyrosine kinase, mediates intracellular signaling mainly through the activation of the common γ chain of several cytokine receptors (13). Mutations inactivating Jak3 functions lead to immunodeficiency (15, 29), and its abnormal activation is associated with different malignancies (15, 16, 30). Previously, we reported regulation of cytoskeletal remodeling and wound repair through Jak3 interactions with the actin-binding protein villin (20, 22). Jak3 also plays an essential role during mucosal homeostasis and intestinal differentiation (17, 21, 22). Interestingly, although Jak3 interactions with the AJ protein β-catenin were essential for mucosal barrier functions and protection from colitis (7), the structural determinants and molecular mechanism of Jak3 interactions with β-catenin were not known. To achieve this, we used previously reported WT and mutants of Jak3 (20) and WT and mutants of β-catenin to characterize Jak3–β-catenin interactions and determine the kinetic parameters of β-catenin trans-phosphorylation by Jak3. Our data showed that β-catenin not only interacted with Jak3 with a *K_d_* of 0.28 μm but was also a direct substrate for Jak3 (Fig. 1). Moreover, Jak3 trans-phosphorylated β-catenin, where the *t*_½_ of β-catenin trans-phosphorylation was lower than that of Jak3 autophosphorylation (Fig. 1) (20), indicating that Jak3 autophosphorylation was rate-limiting during Jak3-β-catenin interactions. We further confirmed these interactions by inhibition studies, where CP-690550 (31) inhibited β-catenin trans-phosphorylation by Jak3.

β-Catenin is a multifunctional protein that facilitates AJ formation through forming a bridge between E-cadherin and α-catenin (26). β-Catenin is also a pleiotropic transcriptional coactivator that facilitates gene expression during Wnt signaling (32). The role of β-catenin in Wnt signaling has been extensively studied, but there is a limited understanding of the tyrosine phosphorylation-mediated regulation of AJ function by β-catenin. Structurally, β-catenin consists of a 130-amino acid NTD, a 550-amino acid central ARM repeat, and a 100-amino acid CTD. Although it has been reported that the NTD contains phosphorylation sites for GSK-β and that the CTD possesses transcriptional activation functions (33), the mechanism of β-catenin interactions with tyrosine kinase in general and Jak3 in particular was not known. Our data show that Jak3 trans-phosphorylated β-catenin in a time-dependent manner, with a *t*_½_ of trans-phosphorylation of 34 s. Previously, we reported that Jak3 phosphorylates the cytoskeletal protein villin (20, 22) and the adapter protein p52ShcA (18, 19). This study shows that Jak3-mediated phosphorylation of p52ShcA precedes the phosphorylation of villin and β-catenin. We speculate that p52ShcA might facilitate recruitment of both the substrates to Jak3 and their dephosphorylation through recruitment of phosphatases (19) in a cyclic process of phosphorylation and dephosphorylation.

Our understanding of the structure–function relationship between Jaks and their interacting partners is limited. Available reports suggest that Jaks bind to their cytokine receptor through the N-terminal FERM domain (34). In Jak3, the JH4-JH7 FERM domain not only interacts with and activates the JH1 kinase domain, but it also interacts with the JH3–JH4 SH2 domain, thereby maintaining a closed conformation in non-phosphorylated Jak3 (20, 31). Our data suggest that both the FERM and kinase domains of Jak3 interact with the NTD domain of β-catenin, where the SH2 domain of Jak3 partially inhibited these interactions (Fig. 2). Consistent with this, our data show that Jak3 phosphorylates three tyrosine residues, *viz.* Tyr^30^,Tyr^64^, and Tyr^86^, in the NTD of β-catenin. Because Jak3 autophosphorylation was rate-limiting in Jak3 phosphorylation of β-catenin, our data show that prior phosphorylation of β-catenin at Tyr^654^ was also necessary for its interaction with autophosphorylated Jak3 and further phosphorylation at the aforementioned residues. Although phosphorylation of Tyr^654^ in β-catenin has been reported by both receptor and non-receptor tyrosine kinases and is associated with nuclear localization (35), breast carcinoma (36), and EMT (23), its dephosphorylation is associated with increased presynaptic vesicular turnover (37). The functional implications of our data using both normal FHC-CRL1813 and transformed IEC HT-29 cl19a cells showed that Jak3 strongly prevented Tyr^654^-phosphorylated, β-catenin–mediated AJ exit through further phosphorylation of β-catenin at Tyr^30^, Tyr^64^, and Tyr^86^, where Jak3-mediated phosphorylation of these tyrosine residues increased the TEER in both cell types (Fig. 3). This was further corroborated by findings from other groups showing that elevated tyrosine kinase activity stabilizes β-catenin–mediated AJs (38, 39). Controlled regulation of cell proliferation and differentiation is essential and requires a coordinated regulation of cell–cell adhesion and gene transcription. Because activated Jak3 interacted with Tyr^654^-phosphorylated β-catenin, not only *in vitro* but also in human cells, our finding using an *in vivo* mouse model confirmed this and showed that Jak3-expressing wild-type mice had junctional localization of Tyr^654^-phosphorylated β-catenin; however, loss of Jak3 resulted in AJ loss of these Tyr^654^-phosphorylated β-catenin in colonic mucosa. Together, these data validate that Jak3 facilitates AJ reinforcement through promoting β-catenin localization to AJs during stimulation of IECs by growth factors. We speculate that, under physiological conditions, a subtle balance between growth factors and cytokine simultaneously activating IECs may coordinate EMT and barrier functions, as reflected by our data (Fig. 4), where Jak3 activation–mediated reinforcement of AJ is due to tyrosine phosphorylation of Tyr^30^, Tyr^64^, and Tyr^86^ of Tyr^654^-phosphorylated β-catenin. The role of IECs is critical for overall human health, as they are in a very hostile environment of a huge concentration of not only immune cells beneath mucosal surfaces but also in the presence of gut microbiota toward luminal sides (40). In such an environment, regulation of EMT is critical not only for normal wound repair by IECs but also for preventing neoplastic transformation and metastasis. As Tyr^654^ phosphorylation of β-catenin is known to promote proliferation, Jak3-mediated phosphorylation of Tyr^30^, Tyr^64^, and Tyr^86^ of Tyr^654^-phosphorylated β-catenin could be critical to put a brake on proliferation, particularly after successful completion of wound repair and transition into redifferentiation, where reinforcement of AJ becomes important. Our data demonstrate that activation of Jak3 could be critical to apply this brake by suppressing EMT and promoting reinforcement of AJs through phosphorylation of the aforementioned three critical tyrosine residues in Tyr^654^-phosphorylated β-catenin, as shown in both in normal and transformed human IECs (Figs. 4 and 5). It is known that forced expression of N-cadherin in an epithelial environment strongly correlates with neoplastic transformation and colonic polyp formation (41). Moreover, in the progression from normal epithelial cells to cancerous cells, colonic polyps represents a transition state, where EMT plays a critical role in this progression, and, in humans, sustained expression of the EMT marker N-cadherin is frequently reported (24, 25) in colonic polyps. The physiopathological implications of our studies show that human colonic polyps not only have significantly reduced Jak3 expression and localization compared with healthy mucosal counterparts but that these polyps also have a significantly elevated level of the EMT marker N-cadherin, indicating that robust Jak3 expression is required for the suppression of EMT and associated colonic polyp formation. Thus, our data from human biopsies of colonic polyps and associated healthy tissues corroborate our *in vitro* and *in vivo* findings, which showed that loss of Jak3 expression was associated with increased expression of EMT markers, particularly in polyps, but not in healthy tissues, where Jak3 expression was normal (Fig. 5). Additionally, our data on molecular simulation and structural comparison using phospho-mimetic approaches validated that Jak3-mediated stabilization of AJs and suppression of EMT were due to Tyr^30^, Tyr^64^, and Tyr^86^ phosphorylation–induced reversal of significantly changed orientation of the β-catenin-NTD that was induced by Tyr^654^ phosphorylation. These might be responsible for the enhanced interaction of β-catenin–NTD with the AJ protein α-catenin, thereby stabilizing AJs and enhancing barrier functions.

Taken together, these results show, for the first time, the structural determinants of Jak3 and β-catenin responsible for their interactions and determine the previously unknown tyrosine residues of β-catenin phosphorylated by Jak3 that not only increase the barrier functions but also inhibit EMT. Thus, this study demonstrates a novel molecular mechanism of Jak3 interactions with β-catenin and their physiological and pathophysiological implications in AJ formation, EMT, and colonic polyps, which, in the future, would have a bigger impact on our understanding of intestinal wound repair, neoplastic transformation of colonic IECs, and cancer metastasis.

## Experimental procedures

### Materials

The deidentified and discarded human colonic biopsy samples used in this study were obtained from CHRISTUS Spohn Health System laboratory. HT-29 Cl-19A, a permanently differentiated homogenous clone derived from human colonic epithelial cells (HT-29) was a kind gift from Dr. A. P. Naren (Cystic Fibrosis Research Center (CFRC), Cincinnati, OH) (42). FHC (CRL-1831^TM^, catalog no. 30-2006) was purchased from the ATCC. IL-2, CP-690550, and EGF were from Sigma); J67242 was from Alfaaesar, and the Pierce BCA protein assay kit was from Thermo Scientific. The following antibodies were used: pTyr^20^ (MP Biomedicals); Jak3 (Invitrogen); β-catenin, p52ShcA, villin, N-cadherin, β-actin, and Shc (Santa Cruz Biotechnology); p654–β-catenin (Abcam); Hif1α (Novus Biologicals); α-catenin and FITC (Sigma); His (Genescript); secondary fluorophore conjugate of anti-mouse FITC (Sigma); and anti-rabbit CY3 (Amersham Biosciences).

### Cell culture, IL-2 and EGF treatment, and stable transfection

Cell maintenance and culture for FHC (ATCC, CRL1831^TM^) were done according to the specification sheet provided by the ATCC. However, treatment and transfection in FHC (ATCC, CRL1831^TM^) were done according to the protocol of the supplier. The methods for HT-29 cell maintenance and treatment have been reported before (18, 22). DNA constructs for pCDNA-HA-β-catenin and mutants were stably transfected into HT-29 cells as reported before (43). Transfected HT-29 Cl-19A cells were grown to confluence, followed by treatment with IL-2 (50 units/ml) (22). For activation of Src kinase, cells were treated with EGF (38), and for inhibition of Src kinase, the Src kinase inhibitor was as reported before (44). For Jak3 inhibition, cells were treated with CP-690550 as reported before (18, 22, 31). PCDNA-HA-β-catenin-WT, β-catenin-HA NTD+ARM, and β-catenin-HA-Y41F,Y6F,Y181F were stably transfected into HT-29 CL19A cells and PCDNA-HA-β-catenin-WT and HA-β-catenin-Y41F,Y6F,Y181F using methods as reported before (18).

### Site-directed mutagenesis, expression and purification of the recombinant proteins, in vitro kinase assay, and protein–protein interaction

Mutations in full-length β-catenin cDNA and the β-catenin NTD were done by site-directed mutagenesis using methods reported previously (45). His-tagged WT and mutant constructs of β-catenin were transformed in *Escherichia coli* BL21 and TKX1 cells to produce the non-phosphorylated and phosphorylated forms of recombinant proteins, respectively, using methods reported previously (43). *In vitro* kinase and pairwise binding assays were performed as reported before (19, 20). Kinetic parameters were determined as reported before (43).

### Measurement of TEER

FHC (ATCC, CRL1831^TM^ cells transiently transfected with pCDNA-HA β-catenin-WT and pCDNA-β-catenin-HA-Y30F,Y64F,Y86F were seeded in the apical compartment of 1.12-cm^2^ Transwell® polyester membrane inserts with 0.4 μm pores (Corning Inc., New York, NY) at a seeding density of 1 × 10^5^ cells/insert and differentiated. Likewise, HT-29 CL19a cells stably transfected with pCDNA-HA β-catenin-WT and pCDNA-β-catenin-HA-Y30F,Y64F,Y86F were grown to confluence. TEER was measured using the Millicell electrical resistance system (Millipore) as reported previously (46, 47) and expressed as ohms per square centimeters, where ohm is the observed value of resistance and square centimeters indicate the surface area of the membrane. The background resistances of the transwell membrane (∼30 ohms/cm^2^) were subtracted from the observed resistance values. Cells without transfection were taken as the control.

### Western blotting (IB), IP, immunofluorescence microscopy (IFM), and immunohistochemistry (IHC)

Protein expression of N-cadherin, Hif1α, β-catenin, shc villin, and β-actin was examined by Western blotting. Cells with or without treatment were washed three times with 1× PBS and lysed with radioimmune precipitation assay lysis buffer that contained a mixture of protease inhibitors. Following quantification of total protein with the BCA® protein assay kit, 80 μg of the whole-cell lysate was denatured in Laemmli buffer at 95 °C for 10 min, resolved on 10–12% SDS-PAGE, and electrotransferred onto nitrocellulose membranes. The membranes were blocked in TBS that contained 0.05% Tween 20 and 1% BSA for 1 h at room temperature. Blots were then incubated with primary antibody followed by secondary antibody for 90 min at room temperature. Membrane-bound secondary antibodies were visualized with a chemiluminescence HRP substrate (Roche Applied Sciences) using a Chemilluminager. The methods for IP and IB using HT-29 CL-19A cell lysates under indicated experimental conditions were reported previously (18). For IFM, cells grown on coverslip sections were air-dried, fixed using paraformaldehyde, and blocked with 0.1% BSA in PBS. The sections were then incubated with primary antibodies for the indicated proteins, followed by incubation with cy3 or Alexa Fluor 488–conjugated secondary antibodies and washing with 1× Dulbecco's phosphate-buffered saline (DPBS) and mounted using Vectashield (Vector Laboratories). For all negative controls, primary antibodies were replaced with a control non-immune IgG at the same concentration. The immunostained slides were visualized using a C1-plus Nikon laser-scanning confocal microscope, and the images were processed using NIS Element software (Nikon^R^). For IHC, the aforementioned sections from frozen tissue blocks were fixed with precooled acetone at 20 °C. Endogenous peroxidase activity was inactivated by incubating the tissue with 0.3% H_2_O_2_. Nonspecific binding was blocked by incubation with 10% fetal bovine serum prior to incubation with primary antibodies. The sections were then incubated with biotinylated secondary antibody followed by staining with the Vectastain ABC kit (Vector Laboratories). The reaction was visualized using 3,3-diaminobenzidine substrate. All experiments were performed at least in triplicate, and representative images are shown.

### FACS analysis

HT-29CL19A cells with or without treatment were trypsinized and seeded in 24-well cell culture–grade plates (Thermo Fisher Scientific, Rochester, NY). To assess the role of Jak 3 in suppressing EMT induced by EGF, the cells were either treated with IL-2 alone (50 units/ml) or in combination with EGF (25) and CP-690550 (18, 23), as mentioned previously. Untreated HT 29 CL 19A cells were taken as a control for the experiment. Similarly, cells were treated either with EGF alone or in combination with a Src kinase inhibitor taken as a negative control. EGF activates Src kinase, phosphorylating β-catenin at Tyr^654^, and CP-690550 inhibits Jak3 activity. The treated cells were detached by using 2 mm EDTA in PBS, washed, and resuspended in 1:100 dilutions of primary antibodies for N-cadherin, Jak 3, p654 β-catenin, β-catenin, and Hif1α in a solution containing PBS with 1% BSA and 0.01% sodium azide for all experimental conditions. A secondary fluorophore conjugate of anti-mouse FITC for N-cadherin, p654 β-catenin, or anti-rabbit CY3 for Jak 3, β-catenin, and Hif 1α was used subsequent to incubation with primary antibody. The experimental samples were incubated at room temperature for 30 min and washed in PBS with 1% BSA and 0.01% sodium azide after incubation with primary and secondary antibodies. Flow cytometric data were acquired in FL 1 (FITC, 533/30) and FL 2 (Cy3, 585/40) channels using a BD Accuri C6 flow cytometer (BD Biosciences) and analyzed using BD Accuri C6 software with appropriate unstained and gating controls. The percentages of cells determined to be positive on the FL1 or FL 2 channels were plotted in the form of a bar graph with Origin 8.6 (Originlab) software and statistically compared for all treatment groups.

### Homology modeling and phosphomimetic simulations of β-catenin protein

Full-length β-catenin protein and its Src-mediated (Tyr^654^) or Jak3-mediated (Tyr^30^, Tyr^64^, and Tyr^86^) phosphomimetic counterparts were modeled using the crystal structure of full-length β-catenin protein reported previously (9) with the Phyre2 server (48). β-Catenin was modeled in intensive modeling mode using the crystal structure (PDB code 2Z6G) as the template. The intensive modeling mode of the Phyre2 homology modeling software combines multiple template modeling and simplifies *ab initio* protein folding simulations to create the full-length model of the protein. To simulate the impact of phosphorylation of tyrosine residues (Tyr^30^, Tyr^64^, and Tyr^86^, and Tyr^654^) of β-catenin, these amino acids were replaced with the phosphomimetic residue Glu. The conformational changes induced in the structure of β-catenin were analyzed by superimposition and energy minimization using CHARMM Forcefield and visualized in Accelerys Discovery Studios (Biovia).

### Data analysis

All data presented are mean (± S.E.) and were analyzed using Microcal Origin^R^ software version 9.2. Differences in the parametric data were evaluated by Student's *t* test. Significance in all tests was set at a 95% or greater confidence level.

## Author contributions

N. K. and J. M. conceived and coordinated the study and wrote the paper. J. M. designed, performed, and analyzed the experiments shown in Figs. 1–5. J. K. D. designed, performed, and analyzed the experiments shown in Figs. 5, *A* and *B*, and 6 and supplemental Figs S1–S3. All authors reviewed the results and approved the final version of the manuscript.

## Supplementary Material

Supplemental Data

## Footnotes

3 The abbreviations used are:

AJ: adherens junction
EMT: epithelial–mesenchymal transition
NTD: N-terminal domain
ARM: armadillo
CTD: C-terminal domain
JH: Jak homology
P: phosphorylated
IEC: intestinal epithelial cell
FHC: fetal human colon
SH: Src homology
TEER: trans-epithelial electrical resistance
IP: immunoprecipitation
IB: immunoblotting
IFM: immunofluorescence microscopy
IHC: immunohistochemistry.
