# Supplementary material for: Janus kinase 3 regulates adherens junctions and epithelial mesenchymal transition through β-catenin
Source: J Biol Chem. 2017 Aug 17;292(40):16406–19. doi: 10.1074/jbc.M117.811802 (PMC5633104; doi:10.1074/jbc.M117.811802)
Supplement: Supplemental Data [file supp_292_40_16406__index.html]

Janus kinase-3 regulates adherens junction and epithelial mesenchymal transition through Beta-catenin — Janus kinase 3 regulates adherens junctions and epithelial mesenchymal transition through β-catenin — Jak3 interactions with β-catenin–NTD suppress EMT — Supplemental Data 

# Janus kinase 3 regulates adherens junctions and epithelial mesenchymal transition through β-catenin

## Supplemental Data

- Supplemental Figures (.pdf, 254 KB) - Supplemental Figures S1, S2, and S3.
