# Supplementary material for: Janus kinase 3 regulates adherens junctions and epithelial mesenchymal transition through β-catenin
Source: J Biol Chem. 2017 Aug 17;292(40):16406–19. doi: 10.1074/jbc.M117.811802 (PMC5633104; doi:10.1074/jbc.M117.811802)
Supplement: Supplemental Data [file 10.1074_M117.811802_jbc.M117.811802-1.pdf]

Supplemental Figures.

Fig. S1-S3. IL-2-mediated activation of Jak3 suppress colonic EMT during EGF-mediated activation of Src. *S1-S2,* HT-29 Cl-19a cells were either unstimulated (control) or stimulated with EGF and/or IL-2 alone or in presence of Src-kinase inhibitor (SRCi) or Jak3-kinase inhibitor (Tof). Flow-cytometry estimation of the cells were performed as detailed under "*Materials and Methods*" section to determine the expression of EMT marker Hif1-α. Note that (*A*) top panels confirm EGF-stimulation of EMT marker Hif1-α whereas the bottom panels show reversal of these effects by IL-2 mediated activation of Jak3. There was a (*B*) ten-fold increase in the expression of EMT marker by EGF that was suppressed almost four-fold by IL-2 activation of Jak3. *A,* Scattered plots are representative (n=3 experiments). *B,* Plots are average of three experiments where ** shows statistically significant difference with control (for EGF) and with IL-2+EGF (for IL-2+EGF+Tof). *S3***,** IHC staining for the localization of Hif1-α in colonic mucosa of WT and Jak3-KO mouse was performed using the protocol described under "*Experimental Procedures*." Red arrow indicates the differences in localization of Hif1-α in absence of Jak3. Please note that lack of Jak3 results in loss of Hif1-α in the epithelial lining of the colonic mucosa. The data shown are representative (*n*=6 per group).

Supplemental Figures

S1

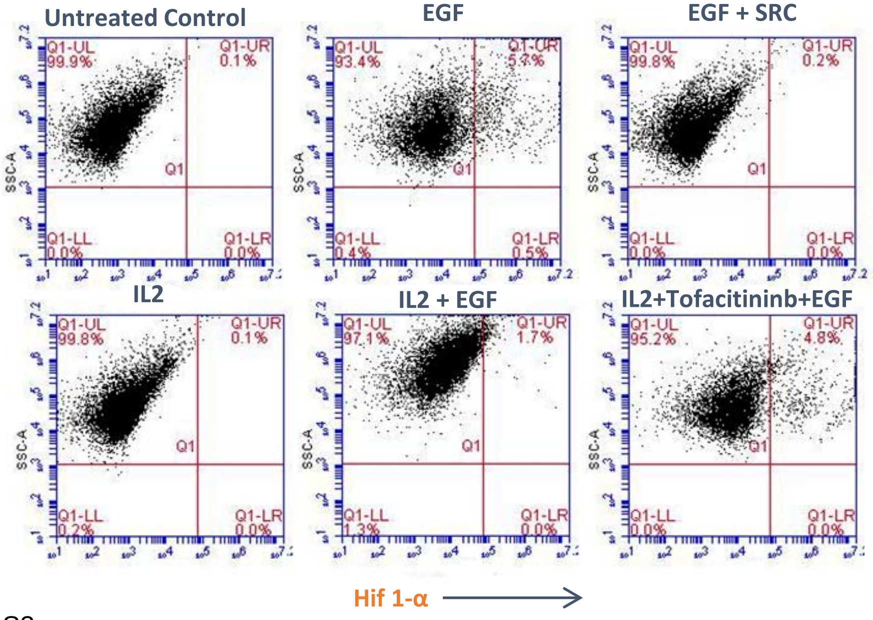

S2

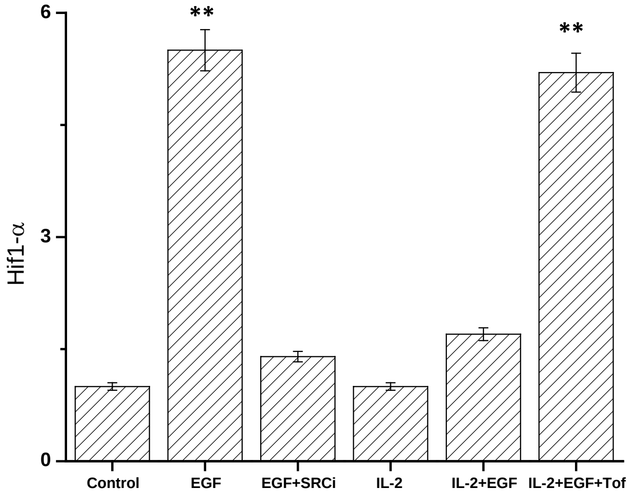

S3

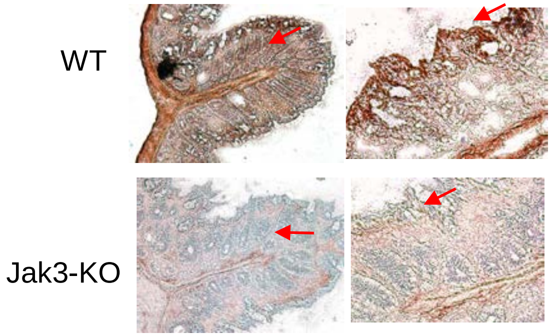

IHC: Hif1α (mouse colon)
